# Lower-Strength Alcohol Products and Public Health

**DOI:** 10.3390/nu15102240

**Published:** 2023-05-09

**Authors:** Peter Anderson

**Affiliations:** Department of Health Promotion, CAPHRI Care and Public Health Research Institute, Maastricht University, 6200 MD Maastricht, The Netherlands; peteranderson.mail@gmail.com

The World Health Organization has called on economic operators to “substitute, whenever possible, higher-alcohol products with no-alcohol and lower-alcohol products in their overall product portfolios, with the goal of decreasing the overall levels of alcohol consumption in populations and consumer groups, while avoiding the circumvention of existing regulations for alcoholic beverages and the targeting of new consumer groups with alcohol marketing, advertising and promotional activities” (see [1]).

As Anderson et al. discuss, lower-strength alcohol products can be split into two groups: (i) specifically produced no-alcohol products; (ii) existing products reformulated to contain less alcohol [1]. Across all main beverage categories (beers, wines, and spirits), alcohol companies are producing both types of products [1,2,3,4].

When considering the public health impact of non-alcoholic products, one of the difficulties relating to monitoring and evaluation is the differing definitions across jurisdictions [5]. To resolve this, Okaru & Lachenmeier propose that zero-alcohol products (0.0% vol) are those products with an alcohol by volume (ABV) of <0.04% (to account for measurement errors), and that no-alcohol products are those with an ABV <0.5% [5].

As reviewed by Anderson et al. [1], and specifically studied using household purchasing data in Spain [2], households that recently bought either no-alcohol beer or wine subsequently reduced their purchases of higher-strength beers and wines, respectively, and of total grams of alcohol; in other words, households substituted purchases of higher-strength products with no-alcohol products. Analyses of British household purchase data revealed similar substitutions (see [1,2]).

Further analyses of Spanish household data found that decreases in the ABV of existing beers and wines had a much greater impact in reducing purchases (in grams) of all alcohol than the relatively small increases in purchases of no-alcohol beers and wines [3]. Analyses of British household purchase data produced similar findings (see [3]). Further, with respect to beer, decreases in prices of no-alcohol beer and increases in prices of higher-strength beers led to decreased purchases of grams of all alcohol; the greatest impact was when prices of higher strength beers increased more steeply with increasing ABV [3].

With respect to the health impact, it has been questioned whether people with severe alcohol problems (commonly called “alcohol use disorder”) safely switch to consuming no-alcohol products. On the one hand, as noted in the review by Anderson et al. [1], survey data from the United Kingdom, collected during 2021, found that two-fifths of respondents who had drunk no- and low- alcohol products within the previous 12 months did so because they were trying to reduce their alcohol consumption, with 7% doing so because they were recovering from “alcohol dependency”. On the other hand, in their review, albeit based on only two to three studies, Caballeria et al. found evidence that although nearly half of patients treated for “alcohol use disorder” used no-alcohol products during social events, such products could lead to increased cravings and an increased desire to drink [6].

Rehm et al. undertook a modeling study to estimate the population-level health impact achieved by the current introduction of no- or low-alcohol beverages in Great Britain and Spain, and the impact of reducing the ABV of all alcohol products by a relative 10% amongst France, Germany, Italy, Poland, Spain, and United Kingdom [7]. They found that the current introduction of no- or low-alcohol beverages in Great Britain and Spain is associated with reductions in overall mortality, but to such a small extent that the reductions at a population level were, to any extent, irrelevant; this is because the consumption of no-alcohol products, although increasing, is only a very small proportion of the volume of consumption of all alcohol products [1]. On the other hand, a relative 10% reduction in the ABV of all alcohol products could have an important population-level impact, reducing overall mortality with a range between 0.42% and 1.26% of all deaths, dependent on the country and whether the consumers are women or men [7].

Through addressing the product, reducing the ABV is only one of several innovative policy approaches. An alternative approach is to reduce serving sizes. The narrative review of Mantzari & Marteau found that reducing the size of servings, glasses, and bottles of wine could reduce wine consumption across populations [8].

Nutt and colleagues proposed an even more novel approach: developing functional drinks that do not use alcohol yet mimic the positive, pro-social effects of alcohol without the associated harm [9].

In conclusion, the introduction of no-alcohol products has health benefits for individual consumers, but very limited benefit at the population level due to very low levels of consumption; reformulation of existing products to contain less alcohol could, if realized across all products with a 10% relative reduction in ABV, have a substantial population-level impact, reducing the harm caused by alcohol. Alcohol producers should respond to the World Health Organization’s call for substitution by reducing the alcoholic strength of all their products forthwith. Governments can promote substitution by increasing taxes on alcohol products, with harmonization of taxes per gram of alcohol and taxes rising per ABV of the product; were the tax rises much steeper at very low levels of ABV, this would also promote increased demand for and consumption of no-alcohol products (see [1,7]).
